# Impact of diabetes mellitus on outcomes of patients with sepsis: an updated systematic review and meta-analysis

**DOI:** 10.1186/s13098-022-00803-2

**Published:** 2022-03-05

**Authors:** Li Jiang, Mengdi Cheng

**Affiliations:** 1 Department of Infectious Diseases, The First People’s Hospital of Wenling, Wenling, 317500 Zhejiang China; 2 Department of Emergency Medicine, The First People’s Hospital of Wenling, Wenling, 317500 Zhejiang China

**Keywords:** Sepsis, Septic shock, Diabetes, Mortality, Acute renal failure, Outcomes, Meta-analysis

## Abstract

**Background:**

The effect of concurrent diabetes on the outcome of sepsis is not conclusively known. A meta-analysis published in 2017 indicated that diabetes did not influence the mortality of patients with sepsis but increased the risk of acute renal injury. In view of publication of several new studies in recent years, there is a need for updated evidence.

**Methods:**

A systematic search was conducted using the PubMed, Scopus, Embase, and Google Scholar databases. Studies that were done in patients with sepsis, were observational in design- either cohort or case–control or analysed retrospective data were considered for inclusion. Statistical analysis was performed using STATA software.

**Results:**

A total of 21 studies were included. The risk of in-hospital mortality (RR 0.98, 95% CI 0.93, 1.04) and mortality at latest follow up i.e., within 90 days of discharge (RR 0.94, 95% CI 0.86, 1.04) among diabetic and non-diabetic subjects was statistically similar. There was an increased risk of in-hospital mortality among those with high blood glucose level at admission (RR 1.45, 95% CI 1.01, 2.09). Among those who were diabetic, the risk of acute renal failure (RR 1.54, 95% CI 1.34, 1.78) was higher than non-diabetics. The risk of respiratory failure, adverse cardiac events, need for additional hospitalization post-discharge and length of hospital stay was similar among diabetics and non-diabetics.

**Conclusions:**

Diabetes is not associated with poor survival outcomes in patients with sepsis but is associated with increased risk of acute renal failure. High blood glucose levels, irrespective of the diabetes status, are associated with increased risk of in-hospital mortality. Findings underscore the need for better evaluation of renal function in diabetic patients with concurrent sepsis.

**Supplementary Information:**

The online version contains supplementary material available at 10.1186/s13098-022-00803-2.

## Introduction

Sepsis is defined as a life-threatening organ dysfunction owing to the dysregulated host response to an infection [1]. Sepsis is associated with more than a tenth of the mortality within hospital [1, 2]. An extension to this is a condition known as septic shock that is defined as sepsis in association with circulatory and metabolic abnormalities [3]. In a recent meta-analysis that included 15 studies, the mortality rates due to sepsis and severe sepsis were documented to be 17% and 26%, respectively [4].

It is interesting to note that around one-fifth of the patients with sepsis have associated diabetes mellitus [5]. Diabetes is a metabolic disorder with rising incidence globally. With changing lifestyle and wide acceptance of Western diets that include consumption of processed foods, the incidence of diabetes is nearing pandemic proportions [6]. Patients with diabetes tend to have an increasing predisposition to develop infection and consequent sepsis [7]. In both type 1 and 2 diabetes, there is an increased blood glucose levels and glycemia-dependent immune response alterations that might influence the pathogenesis and outcome of sepsis. Preclinical studies indicate that presence of diabetes influences several components of the innate immune system and exerts an inhibitory effect on the adaptive immune system [8–10]. Diabetes, particularly type 2, results in protracted inflammation, suppression of immune response, and significant morbidity due to infections. In diabetes, there is an activation of inflammatory pathway through activation of toll like receptors such as TLR2 and TLR4 as well as indirect activation through TLR signalling [11, 12].

There has been immense reduction in mortality due to sepsis owing to the advancement in medical treatment and nursing. However, the co-association of sepsis with diabetes is still a considerable medical problem. It is still not conclusively known in what ways the presence of diabetes influences the outcomes of sepsis. In a meta-analysis published in 2017, Wang et al. [13] assessed the impact of diabetes on outcomes of sepsis and included 10 studies. This review concluded that presence of diabetes did not influence the outcome of patients with sepsis; however, the risk of acute renal injury is sufficiently increased in patients with diabetes. In view of publication of several new studies in recent years there is a need for updated evidence. Hence, the purpose of this review was to conduct a thorough literature search and present updated pooled evidence on the impact of diabetes on outcomes of sepsis. The primary outcome of interest was mortality. Other secondary outcomes of interest were complication rates, length of hospital stay and additional need for hospitalization post-discharge.

## Materials and methods

### Search strategy

The study processes were in compliance with PRISMA (Preferred Reporting Items for Systematic Reviews and Meta-analyses) guidelines [14]. A systematic search of English-language publications was conducted via PubMed, Scopus, Embase and Google academic databases for studies published prior to 20th August 2021. Both medical subject heading (MeSH) terminology and free text words were employed (Additional file 1: Table S1). The literature search aimed at identifying studies that examined the association between diabetes status and/or blood glucose levels upon hospital admission with outcomes of interest in patients with sepsis. We registered the study on PROSPERO (CRD42021273785).

### Selection criteria and methods

Search results were listed, duplicates removed, and then two subject experts screened titles and abstracts for study suitability. After this, remaining studies underwent full-text review. Any disagreements regarding inclusion status were resolved through group discussions. Only those studies were included in the meta-analysis that fulfilled the inclusion criteria. In order to identify additional literature, the reference list of the included studies was also reviewed.

#### Inclusion criteria

Studies that were done in patients with sepsis, were observational in design- either cohort or case–control or analysed retrospective data were considered for inclusion. Studies should have examined the outcomes among patients with sepsis based on diabetes status.

#### Exclusion criteria

Case reports and reviews were excluded. Furthermore, studies that did not provide data on outcomes of interest or did not provide comparative findings based on diabetic status were excluded.

### Data extraction and quality assessment

Relevant data was extracted from studies that met inclusion criteria using a pre-determined guide sheet. Extracted data included study identifiers (author names, study year), study setting, study design, subject characteristics, overall sample size, and main findings. Study quality was assessed via the Newcastle–Ottawa Quality Assessment Scale [15].

### Statistical analysis

This meta-analysis was conducted using STATA version 16.0 and reported effect sizes as pooled relative risk (RR) for categorical outcomes and weighted mean difference (WMD) for continuous outcomes. A subgroup analysis was conducted in order to document the effect of diabetes on patients with all stages of sepsis and in those with severe sepsis and/or septic shock. We also analysed and documented the association of blood glucose levels, irrespective of the diabetes status, at the time of hospital admission with the outcomes. All effect sizes were reported along with 95% confidence intervals (CI). I^2^ was used to indicate heterogeneity. If I^2^ exceeded 40%, a random effects model was used [16]. P values under 0.05 indicated statistical significance. Egger’s test was employed to examine publication bias.

## Results

### Study selection, characteristics, and quality

**Fig. 1:**
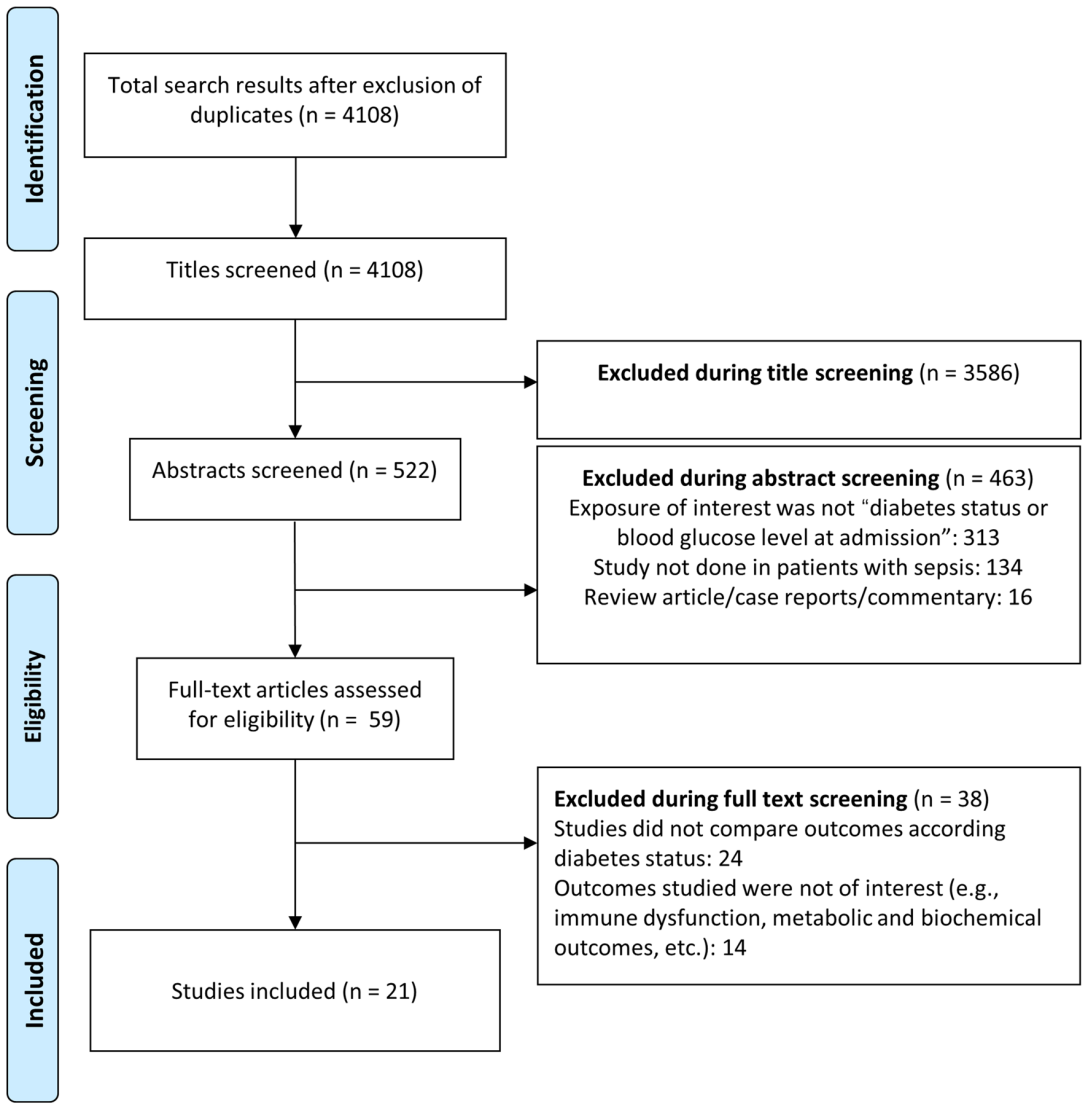
Selection process of the studies included in the review

Database screening yielded 4108 unique citations (Fig. 1). Title screening resulted in the removal of 3586 papers. Of the remaining 522, 463 were excluded after abstract review, with a further 38 excluded after full-text review, leaving 21 for inclusion in the final meta-analysis ([17–37], Table 1). Of these, 14 studies were based on analysis of retrospective data whereas seven studies were prospective in design. Five studies were conducted in USA and three in Greece. Two studies each were done in Spain and Taiwan. There were two multicentric studies and one study each was done in China, Israel, Netherlands, Japan, Singapore, France and South Korea. In 10 studies, the patient population had severe sepsis and/or septic shock and in the remaining 11 studies, the included patient population had varied stages of sepsis. The included studies majorly reported on in-hospital mortality and some studies, additionally reported mortality at around 30- and 90-days post-hospital discharge. The included studies were of good quality (Additional file 1: Tables S2 and S3).

**Table 1:** Characteristics of the studies included in the meta-analysis

Author (year of publication)	Study design	Country	Participant characteristics	Sample size	Key outcomes
Zohar et al. (2021) [17]	Analysis of retrospective data	Israel	Patients with community onset sepsis; median age of 67 years; 56% females; majority with respiratory (36%) or urinary tract (24%) infections; 29% with severe sepsis/septic shock	1527 (diabetes, DM: 469; non-diabetes, non-DM: 1058)	Diabetes vs. no diabetes
					Mortality (in-hospital): RR 1.21 (95% CI 0.80, 1.71)
					Mortality at 30 days post-discharge: RR 1.10 (95% CI 0.79, 1.54)
					Mortality at 90 days post-discharge: RR 1.13 (95% CI 0.86, 1.49)
					Functional deterioration: RR 1.10 (95% CI 0.76, 1.67)
					Discharge to home: RR 1.15 (95% CI 0.65, 2.0)
					Additional need for hospitalization: RR 1.14 (95% CI 0.84, 1.54)
					Acute renal failure: RR 2.48 (95% CI 1.93, 3.19)
					Respiratory failure: RR 1.78 (95% CI 1.21, 2.60)
					Blood glucose level at admission (> 200 mg/dl vs. < 200 mg/dl)
					Mortality (in-hospital): RR 1.48 (95% CI 1.02, 2.16)
					Mortality at 30 days post-discharge: RR 1.80 (95% CI 1.12, 2.58)
					Mortality at 90 days post-discharge: RR 1.68 (95% CI 1.24, 2.27)
Vught et al. (2017) [18]	Analysis of retrospective data	Netherlands	Patients with sepsis; median age of 70 years; 55% females; majority with respiratory (40%), gastrointestinal (25%) infections	41,492 (DM: 8085; Non-DM: 33,407)	Diabetes vs. no diabetes
					Mortality (in-hospital): RR 1.14 (95% CI 1.07, 1.21)
					Mortality at 90 days post-discharge: RR 1.09 (95% CI 0.72, 1.66)
					Acute renal failure: RR 1.32 (95% CI 1.23, 1.42)
					Respiratory failure: RR 1.41 (95% CI 1.33, 1.49)
					Adverse cardiac event: RR 1.11 (95% CI 1.03, 1.19)
					Additional need for hospitalization: RR 1.33 (95% CI 1.19, 1.48)
					Length of hospitalization (days; Mean): 15 (3.3) vs. 15 (3.1)
					Blood glucose level at admission (> 200 mg/dl vs. < 200 mg/dl)
					Mortality at 90 days post-discharge: RR 0.97 (95% CI 0.90, 1.05)
Chao et al. (2017) [19]	Analysis of retrospective data	Taiwan	Patients with median age of 66 years; > 50% males; majority with septic shock (70%); bacteraemia (20%)	6156 (DM: 3594; Non-DM: 2562)	Diabetes vs. no diabetes
					Mortality (in-hospital): RR 0.83 (95% CI 0.65, 0.99)
					Additional need for hospitalization: RR 0.83 (95% CI 0.68, 1.02)
					Acute renal failure: RR 1.78 (95% CI 1.52, 2.10)
					Blood glucose level at admission (> 200 mg/dl vs. < 200 mg/dl)
					Mortality (in-hospital): RR 1.83 (95% CI 1.30, 2.80)
Sathananthan et al. (2019) [20]	Analysis of retrospective cohort data	USA	Majority with age > 60 years; > 55% males; majority with severe sepsis (> 80%)	1698 (DM: 508; non-DM: 1190)	Diabetes vs. no diabetes
					Mortality at 30 days post-admission: RR 1.00 (95% CI 0.81, 1.25)
					Acute renal failure: RR 1.53 (95% CI 1.23, 1.90)
					Respiratory failure: RR 1.16 (95% CI 0.92, 1.48)
					Adverse cardiac event: RR 1.09 (95% CI 0.88, 1.34)
					Length of hospitalization (days; Mean): 13 (3.2) vs. 13 (2.8)
					Discharge to home: RR 0.97 (95% CI 0.76, 1.19)
					Blood glucose level at admission (> 180 mg/dl vs. < 180 mg/dl)
					Mortality at 30 days post-admission: RR 0.82 (95% CI 0.62, 1.07)
Kushimoto et al. (2020) [21]	Retrospective analysis of prospectively collective data	Japan	Mean age of 73 years; 60% males; Majority with pulmonary (31%) and gastrointestinal infection (26%); 63% with septic shock	1127 (DM: 261; non-DM: 866)	Diabetes vs. no diabetes
					In-hospital mortality: RR 1.32 (95% CI 0.96, 1.81)
					Blood glucose level at admission (> 180 mg/dl vs. < 180 mg/dl)
					Mortality (in-hospital): RR 0.72 (95% CI 0.51, 1.00)
					Mortality at 30 days post-discharge: RR 0.90 (95% CI 0.64, 1.27)
					Discharge to home: RR 0.86 (95% CI 0.62, 1.17)
					Length of hospitalization (days; Mean): 24 (6.3) vs. 23.8 (5.7)
Lin et al. (2021) [22]	Retrospective analysis data	China	Mean age of 66.7 years; 51% males; Majority with bloodstream (44%) and urinary tract infection (21%)	5774 (2887 in each of the DM and non-DM groups)	Diabetes vs. no diabetes
					Mortality (in-hospital): RR 0.73 (95% CI 0.62, 0.87)
					Mortality at 28 days post-discharge: RR 0.86 (95% CI 0.77, 0.97)
					Length of hospitalization (days; Mean): 10.82 (2.1) vs. 10.62 (2.3)
					Acute renal failure: RR 0.97 (95% CI 0.77, 1.22)
					Respiratory failure: RR 0.98 (95% CI 0.88, 1.08)
					Blood glucose level at admission (> 200 mg/dl vs. < 200 mg/dl)
					Mortality at 28 days post-discharge: RR 0.49 (95% CI 0.38, 0.64)
Akinosoglou et al. (2021) [23]	Retrospective analysis of data	Greece	Mean age of 76.2 years; 45% males; majority (76%) with either frank sepsis and/or septic shock	812 (406 in each of the DM and non-DM groups)	Diabetes vs. no diabetes
					Mortality at 28 days post-discharge: RR 1.04 (95% CI 0.75, 1.44)
					Adverse cardiac event: RR 1.16 (95% CI 0.55, 2.47)
Moss et al. (2000) [24]	Prospective cohort study	USA	Mean age of around 54 years; majority of the diabetics were females (62%); Majority with respiratory or urinary tract infections; majority had septic shock	113 (DM: 32; non-DM: 81)	Diabetes vs. no diabetes
					Mortality (in-hospital): RR 0.67 (95% CI 0.36, 1.23)
					Respiratory failure: RR 0.53 (95% CI 0.28, 1.01)
Moutzouri et al. (2008) [25]	Prospective cohort study	Greece	Mean age of around 60 years; around 50% were females; majority with urinary tract infections; most had severe sepsis/septic shock	64 (DM: 24; non-DM: 40)	Diabetes vs. no diabetes
					Mortality (in-hospital): RR 1.30 (95% CI 0.56, 3.03)
Stegenga et al. (2010) [26]	Retrospective study	Multicentric study	Mean age of 60.6 years; around 58% were males; Most had severe sepsis/septic shock	830 (DM: 188; non-DM: 642)	Diabetes vs. no diabetes
					Mortality at 28 days post-discharge: RR 1.03 (95% CI 0.81, 1.31)
					Mortality at 90 days post-discharge: RR 1.00 (95% CI 0.71, 1.41)
					Blood glucose level at admission (> 200 mg/dl vs. < 200 mg/dl)
					Mortality at 28 days post-discharge: RR 2.02 (95% CI 1.28, 3.18)
					Mortality at 90 days post-discharge: RR 2.08 (95% CI 1.31, 3.28)
Schuetz et al. (2011) [27]	Retrospective study	USA	Mean age of 59 years; around 49% were males; around one-third (37%) had severe sepsis/septic shock	7754 (DM: 1844; non-DM: 5910)	Diabetes vs. no diabetes
					Mortality (in-hospital): RR 0.85 (95% CI 0.71, 1.01)
					Blood glucose level at admission (> 200 mg/dl vs. < 200 mg/dl)
					Mortality (in-hospital): RR 2.05 (95% CI 1.40, 2.99)
Yang et al. (2011) [28]	Retrospective study	Singapore	Mean age of 60 years; around 50% were males; majority with respiratory, urinary tract or gastrointestinal infections	9221 (DM: 2943; non-DM: 6278)	Diabetes vs. no diabetes
					Mortality (in-hospital): RR 0.96 (95% CI 0.88, 1.05)
					Length of hospitalization (days; Mean): 12.1 (11.1) vs. 12.2 (14.2)
					Acute renal failure: RR 1.91 (95% CI 1.80, 2.02)
					Respiratory failure: RR 0.81 (95% CI 0.71, 0.93)
					Adverse cardiac event: RR 0.94 (95% CI 0.84, 1.05)
Schuetz et al. (2012) [29]	Prospective cohort	USA	Mean age of 60 years; around 48% were females; majority with pneumonia (22%) or skin/soft tissue infection (27%) or urinary tract infections (11%)	1849 (DM: 539; non-DM: 1310)	Diabetes vs. no diabetes
					Mortality (in-hospital): RR 0.95 (95% CI 0.48, 1.90)
					Length of hospitalization (days; Mean): 6.28 (6.93) vs. 5.67 (6.91)
					Blood glucose level at admission (> 180 mg/dl vs. < 180 mg/dl)
					Mortality (in-hospital): RR 1.48 (95% CI 0.86, 3.83)
Chang et al. (2012) [30]	Prospective cohort	Taiwan	Mean age of 67 years; > 50% males; majority with pneumonia (43%) or gastrointestinal infection (34%) or urinary tract infections (26%); majority with severe sepsis/septic shock	16,497 (DM: 4573; non-DM: 11,924)	Diabetes vs. no diabetes
					Mortality (in-hospital): RR 1.00 (95% CI 0.94, 1.07)
					Length of hospitalization (days; Mean): 23.85 (33.52) vs. 23.72 (44.93)
					Acute renal failure: RR 1.54 (95% CI 1.44, 1.63)
					Respiratory failure: RR 0.96 (95% CI 0.94, 0.97)
					Adverse cardiac event: RR 0.98 (95% CI 0.93, 1.03)
Al-Dorzi et al. (2012) [31]	Retrospective cohort	Multicentric (Canada, USA and Saudi Arabia)	Subject age > 60 years; around 45% females; Majority with pneumonia or gastrointestinal infection or soft tissue infections; majority with severe sepsis/septic shock	8670 (DM: 2289; non-DM: 6389)	Diabetes vs. no diabetes
					Mortality (in-hospital): RR 0.96 (95% CI 0.92, 1.01)
Venot et al. (2015) [32]	Prospective cohort	France	Mean age of 67 years; > 60% males; majority with severe sepsis/septic shock	1064 (DM: 318; non-DM: 746)	Diabetes vs. no diabetes
					Mortality (in-hospital): RR 1.32 (95% CI 1.00, 1.74)
					Length of hospitalization (days; Mean): 9.1 (8.5) vs. 16.8 (17.5)
					Acute renal failure: RR 1.30 (95% CI 1.22, 1.38)
De Miguel et al. (2015) [33]	Retrospective cohort	Spain	Mean age of 72 years; > 55% males; majority one or more organ failure	217,280 (DM: 50,611; non-DM: 166,669)	Diabetes vs. no diabetes
					Mortality (in-hospital): RR 0.97 (95% CI 0.96, 0.98)
					Length of hospitalization (days; Mean): 10 (13) vs. 12 (18)
Kim et al. (2014) [34]	Prospective cohort	South Korea	Median age of 69 years; all subjects were females; around 50% had bacteraemia; all had complicated urinary tract infection/pyelonephritis	775 (DM: 246; non-DM: 529)	Diabetes vs. no diabetes
					Mortality (in-hospital): RR 1.19 (95% CI 0.40, 3.62)
					Length of hospitalization (days; Mean): 9 (1.2) vs. 7 (1.67)
Kofteridis et al. (2009) [35]	Retrospective review of records	Greece	Majority with age above 65 years; 35% males; around 20% had bacteraemia; all had pyelonephritis	206 (DM: 88; non-DM: 118)	Diabetes vs. no diabetes
					Mortality (in-hospital): RR 5.47 (95% CI 1.48, 20.1)
					Length of hospitalization (days; Mean): 10 (4.1) vs. 7 (3.8)
					Acute renal failure: RR 1.63 (95% CI 0.84, 3.19)
Peralta et al. (2009) [36]	Retrospective cohort	Spain	Majority with age above 70 years; 50% males; around 15% had septic shock; foci of infection was majorly urinary tract (50%) and gastrointestinal tract (20%)	1112 (DM: 181; non-DM: 931)	Diabetes vs. no diabetes
					Mortality (in-hospital): RR 1.13 (95% CI 0.67, 1.90)
					Length of hospitalization (days; Mean): 13.3 (12) vs. 13.9 (15)
McAlister et al. (2005) [37]	Prospective cohort	USA	Majority with age above 70 years; around 50% males; 15% were nursing home residents, 49% were taking at least four prescribed medications	2471 (DM: 824; non-DM: 1647)	Diabetes vs. no diabetes
					Mortality (in-hospital): RR 1.00 (95% CI 0.69, 1.45)
					Length of hospitalization (days; Mean): 8 (2) vs. 6.67 (1.33)
					Blood glucose level at admission (> 200 mg/dl vs. < 200 mg/dl)
					Mortality (in-hospital): RR 1.69 (95% CI 0.97, 2.94)

### Diabetes status, hyperglycaemia and mortality in patients with sepsis

**Fig. 2:**
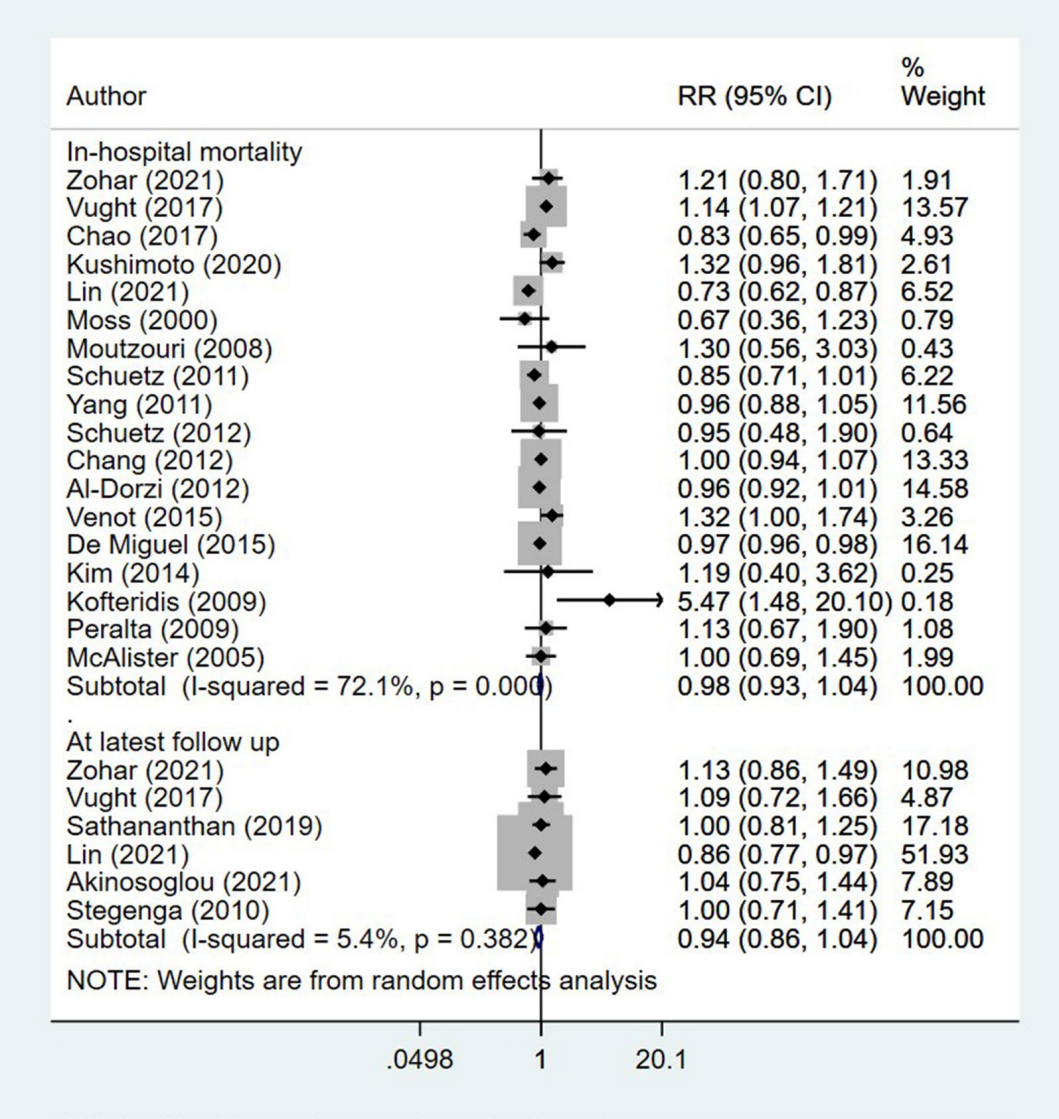
Association of diabetes status with mortality in patients with sepsis

The risk of in-hospital mortality among diabetic and non-diabetic subjects was statistically similar (RR 0.98, 95% CI 0.93, 1.04; I^2^ = 72.1%, N = 18) (Fig. 2). There were no differences in the risk of mortality at the latest follow up among both the diabetics and non-diabetics (RR 0.94, 95% CI 0.86, 1.04; I^2^ = 5.4%, N = 6) (Fig. 2). Egger’s test did not indicate the presence of publication bias (P = 0.23 for in-hospital mortality; P = 0.49 for mortality at latest follow up).

**Fig. 3:**
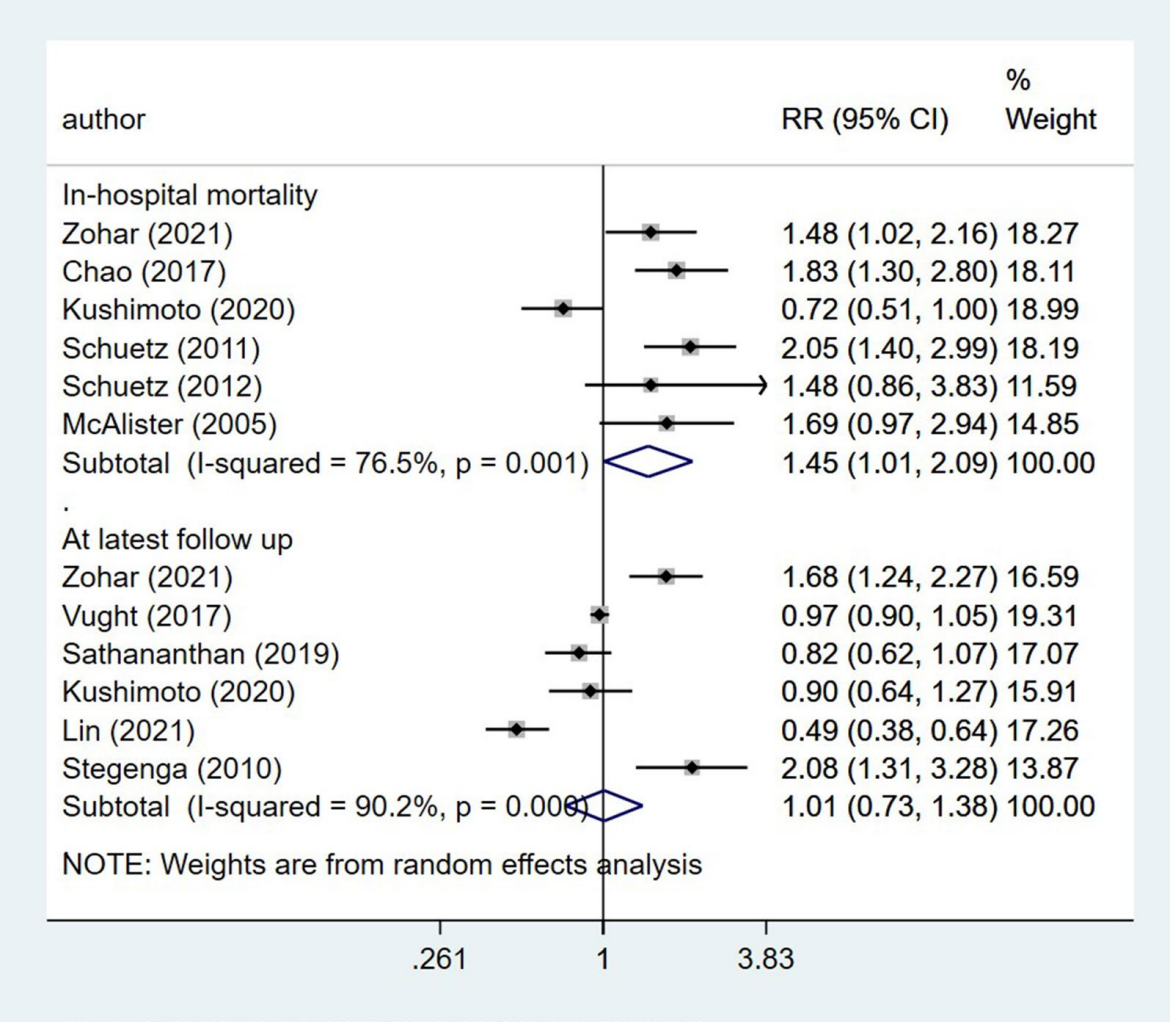
Association of blood glucose level at admission with mortality in patients with sepsis

In the subgroup analysis, the risk of in-hospital mortality and mortality at latest follow up was similar among patients with severe sepsis and those with sepsis of all stages (Table 2). There was an increased risk of in-hospital mortality among those with high blood glucose level at admission (> 180 or > 200 mg/dl) (RR 1.45, 95% CI 1.01, 2.09; I^2^ = 76.5%, N = 6) (Fig. 3). However, at latest follow up, this association was not significant (RR 1.01, 95% CI 0.73, 1.38; I^2^ = 90.2%, N = 6).

**Table 2:** Findings of the subgroup analysis

Outcomes of interest	Severe sepsis	Sepsis of all stage
	Pooled relative risk (RR) with 95% CI	
In-hospital mortality	1.00 (0.91, 1.09)	0.97 (0.89, 1.07)
	N = 7; I^2^ = 54.6%	N = 11; I^2^ = 79.0%
Mortality at latest follow up	1.01 (0.86, 1.18)	0.97 (0.79, 1.19)
	N = 3; I^2^ = 0.0%	N = 3; I^2^ = 50.2%
Acute renal failure	1.51 (1.32, 1.72)	1.57 (1.20, 2.07)
	N = 4; I^2^ = 86.4%	N = 5; I^2^ = 95.7%
Respiratory failure	0.96 (0.77, 1.21)	1.16 (0.85, 1.57)
	N = 3; I^2^ = 65.1%	N = 4; I^2^ = 96.3%
Adverse cardiac event	0.99 (0.94, 1.04)	1.03 (0.87, 1.21)
	N = 3; I^2^ = 0.0%	N = 2; I^2^ = 83.4%

### Diabetes status and risk of complications in patients with sepsis

**Fig. 4:**
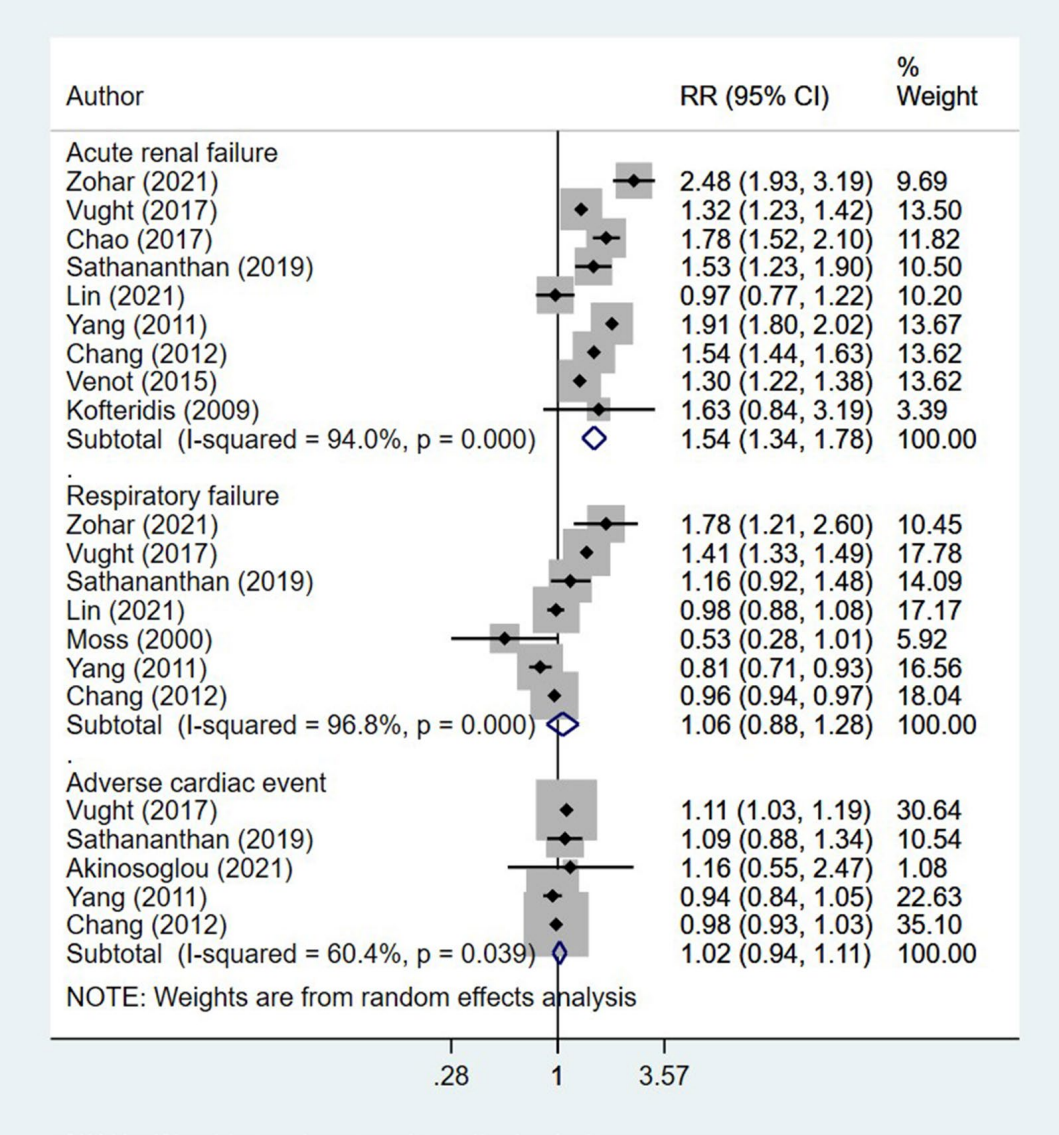
Association of diabetic status with risk of complications in patients with sepsis

Among those who were diabetic, the risk of acute renal failure (RR 1.54, 95% CI 1.34, 1.78; I^2^ = 94.0%, N = 9) was higher than non-diabetics (Fig. 4). For other complications such as respiratory failure (RR 1.06, 95% CI 0.88, 1.28; I^2^ = 96.8%, N = 7) and adverse cardiac events (RR 1.02, 95% CI 0.94, 1.11; I^2^ = 60.4%, N = 5), the risk was similar among diabetics and non-diabetics (Fig. 4). Egger’s test did not indicate the presence of publication bias (P = 0.81 for acute renal failure; P = 0.34 for respiratory failure and P = 0.64 for adverse cardiac event).

In the subgroup analysis, the increased risk of acute renal failure was observed in both patients with severe sepsis (RR 1.51, 95% CI 1.32, 1.72; I^2^ = 86.4%, N = 4) and sepsis of all stages (RR 1.57, 95% CI 1.20, 2.07; I^2^ = 95.7%, N = 5) (Table 2). The risk of respiratory failure and adverse cardiac event with diabetic status was insignificant among the patients in both groups (i.e., severe sepsis and sepsis of all stages).

### Diabetes status and other outcomes of interest in patients with sepsis

**Fig. 5:**
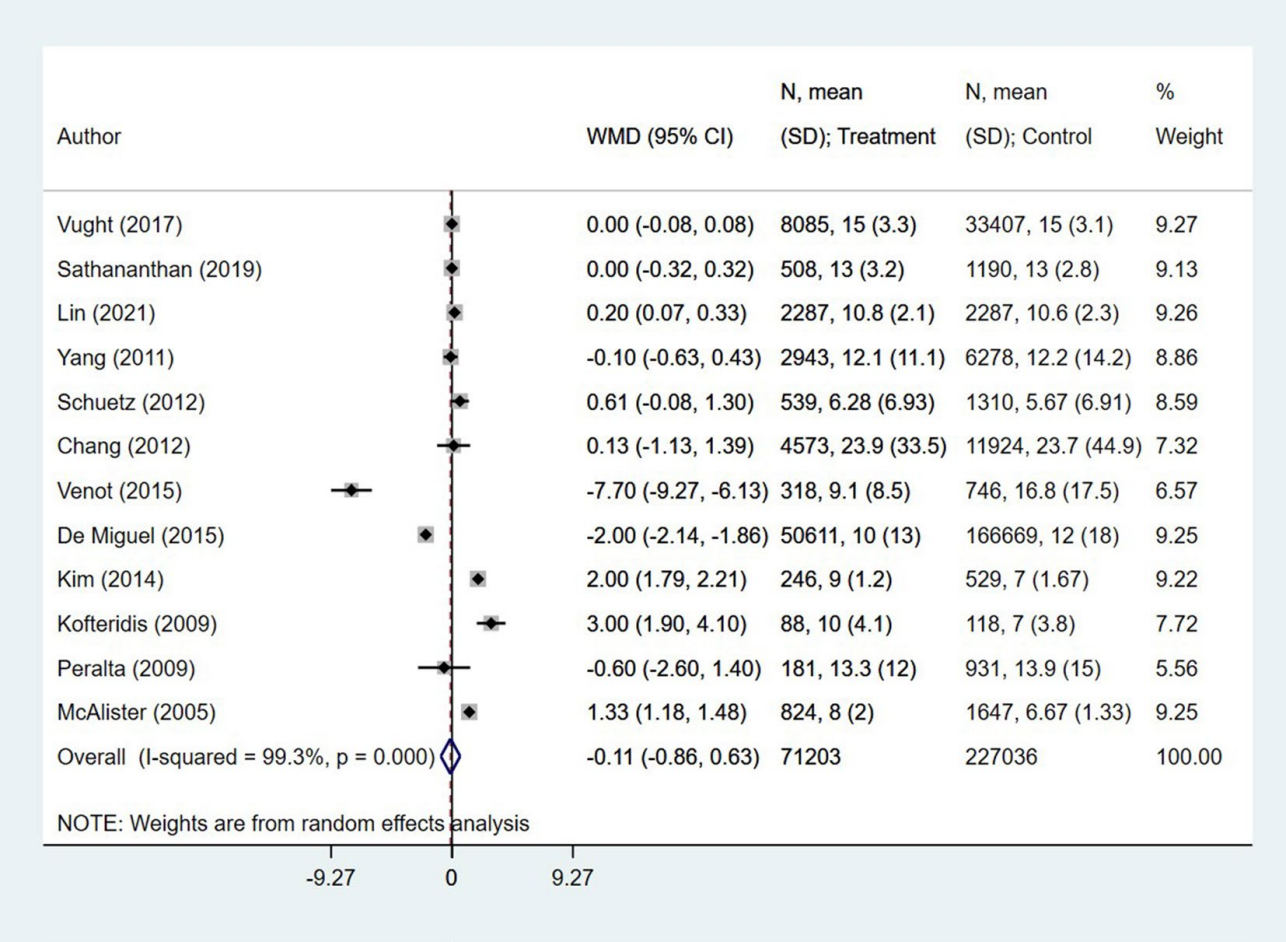
Association of diabetic status with length of hospitalization in patients with sepsis

**Fig. 6:**
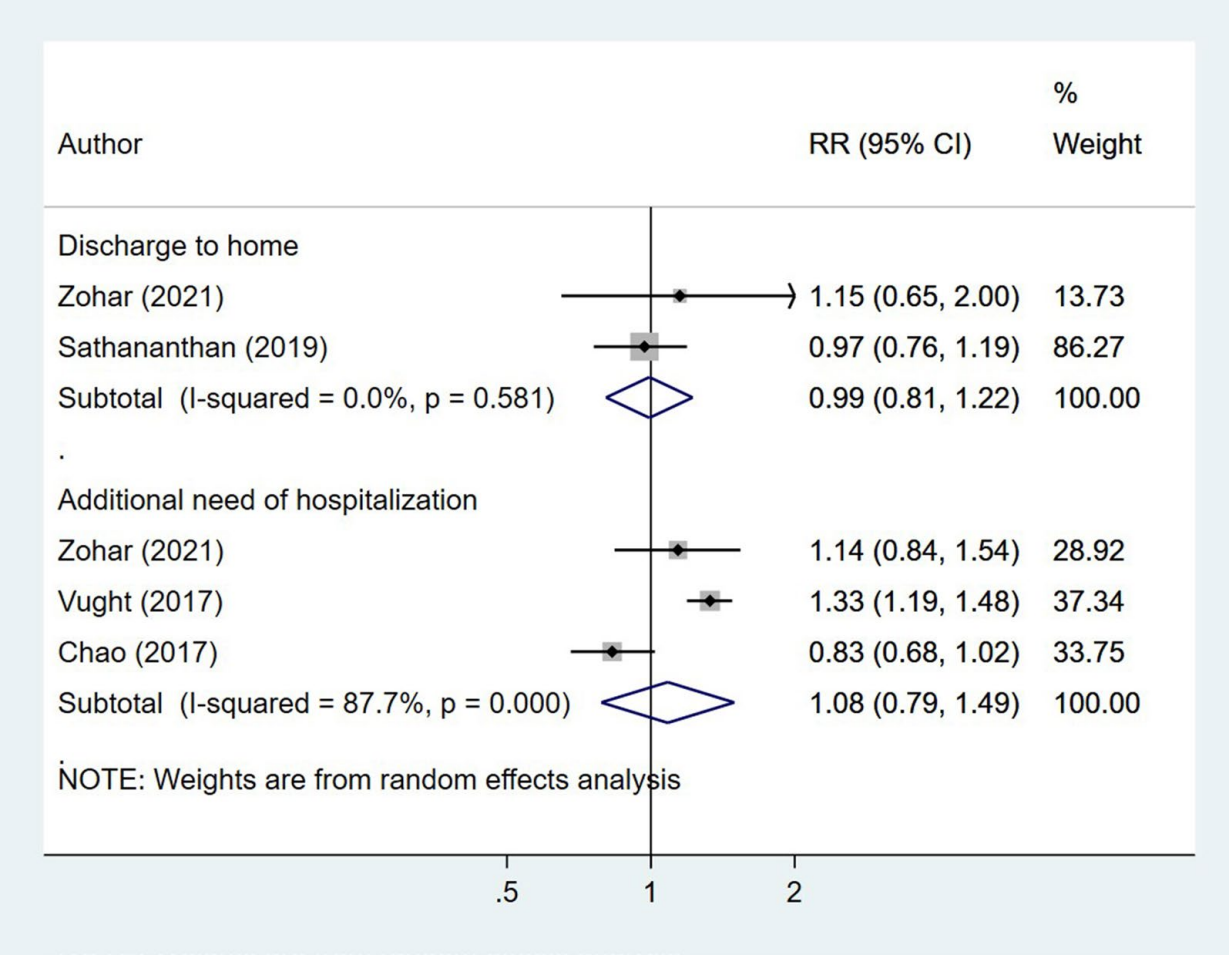
Association of diabetic status with risk of discharge to home and additional need for hospitalization in patients with sepsis

The length of hospital stay (in days) was similar among diabetic and non-diabetics (WMD − 0.11, 95% CI − 0.86, 0.63; I^2^ = 99.3%, N = 12) (Fig. 5). Similarly, the relative risk of additional need for hospitalization (RR 1.57, 95% CI 1.20, 2.07; I^2^ = 95.7%, N = 5) and discharge to home (RR 1.57, 95% CI 1.20, 2.07; I^2^ = 95.7%, N = 5) was similar in both patient groups (Fig. 6). Egger’s test did not indicate the presence of publication bias (P = 0.18 for length of hospital stay; P = 0.39 for additional need for hospitalization and P = 0.48 for discharge to home).

## Discussion

Diabetes adversely impacts the immunological responses of the host. Increased blood glucose levels tend to hamper the function of polymorphonuclear leukocytes (PMN) by lowering the membrane fluidity, thereby resulting in reduced phagocytosis, intracellular killing and sub-optimal migration and chemotaxis [38–40]. Studies have indicated that those with diabetes have multiple downregulated miRNAs encompassing diverse signalling pathways, including MAPK signalling pathway, hematopoietic cell lineage and Fc gamma R-mediated phagocytosis [2, 3, 41]. The present meta-analysis was conducted to present an updated pooled evidence on the association of diabetes mellitus with outcomes in patients with sepsis. The review, through pooling of findings from 21 studies, found that the risk of in-hospital mortality and mortality until 3 months post-discharge among diabetic and non-diabetic subjects was statistically similar. There was an increased risk of in-hospital mortality among those with high blood glucose level at admission, irrespective of the diabetes status. Among those who were diabetic, the risk of acute renal failure was higher than non-diabetics whereas the risk were similar for respiratory failure and adverse cardiac events. The length of hospital stays (in days) and the risk for additional need for hospitalization, post-discharge was similar among diabetic and non-diabetics. Overall, the findings of this meta-analysis do not suggest an increased risk of short-term mortality among patients with sepsis and concurrent diabetes. However, they do point towards the need for maintaining optimal blood glucose levels.

The findings of this meta-analysis are similar to the earlier meta-analysis on this issue by Wang et al. [13]. Both the meta-analysis suggest that presence of diabetes does not increase the risk of mortality in patients with sepsis. In the earlier review, presence of diabetes was associated with slightly reduced risk of in-hospital mortality (pooled relative risk of 0.97, 95% confidence intervals of 0.96–0.98) but in the present review, there were no significant differences in the risk of mortality. This could be more of an analysis issue. In the earlier review, majority of the decrease in mortality estimate was driven by one study (De Miguel et al. [33]; 88.2% weightage) [32]. The increased risk of acute renal failure amongst diabetics has been documented in both the current and the previous meta-analysis. It is well known that diabetes impairs renal function but the underlying pathophysiology is not conclusively known. It is unclear whether the damage is because of the consistent hyperglycaemia milieu, or it results from the end organ damage due to atherosclerosis. There are suggestions that hyperglycaemia can lead to increased activation of NF-kappa B, TGF-*β* and oxidant levels and this in turn, leads to renal damage [42, 43].

The existing evidence is mixed with regards to the effect of acute hyperglycaemia on risk of mortality during an event of sepsis. Acute hyperglycaemia has been documented to be an independent risk factor for mortality in critically ill patients with sepsis [44, 45]. There are also studies that indicate that the effect of hyperglycaemia is modified by the concurrent presence or absence of diabetes [46, 47]. Usually in diabetics, the blood glucose levels remain high and therefore, they are better able to tolerate effects of short-term hyperglycaemia during sepsis. On the other hand, increased blood glucose levels during sepsis in a non-diabetic patient leads to massive increase in inflammatory cytokine level and organ damage. In our meta-analysis, we found that hyperglycaemia was associated with increased risk of in-hospital mortality but not with mortality at latest follow up post-discharge. More research is required to conclusively understand the effect of short-term increased blood glucose levels on the outcomes of sepsis. Research is also warranted on the role of glycosylated hemoglobin (HbA1c) and glycated albumin in predicting the outcomes of sepsis. There is evidence to suggest that the predictive ability of glycated hemoglobin for complications in patients with chronic renal failure is reduced [48, 49]. In such circumstances, glycated albumin could prove reliable as a biomarker for predicting and stratifying the risk linked to multiorgan metabolic alterations [48, 49]. Despite the findings of this review that presence of diabetes in patients with sepsis does not significantly increase the risk of adverse cardiac events, it should be noted that diabetes in itself is a strong risk factor for cardiac complication and a comprehensive assessment of indicators for diabetes control and early markers of cardiac injury is warranted. There have been recent studies that have explored a variety of biomarkers such as galectin-3, troponin-I and heart-type fatty acid binding protein (H-FABP) [50–52]. More such important and groundbreaking studies are required to provide a comprehensive scientific knowledge in this respect.

This is the most updated evidence documenting the effect of diabetes on mortality and other outcomes among patients with sepsis. The included studies are from a wide geography and therefore, the findings are applicable to a large setting. There are some limitations as well. First, majority of the included studies were retrospective data based and therefore, the possibility of important confounders not being adjusted in the analysis cannot be ruled out. Due to the lack of studies reporting on the management protocols for the sepsis, the meta-analysis was not able to ascertain the treatment effects on the outcomes considered. It would have been preferable if the analysis would have looked at the association of glycaemic control (using HbA1c) with outcomes, but such an analysis could not be undertaken as the studies did not report on this.

## Conclusion

Based on pooling of observational studies, our meta-analysis shows that diabetes is not associated with poor survival outcomes in patients with sepsis but is associated with increased risk of acute renal failure. High blood glucose levels (those > 180 mg/dl), irrespective of the diabetes status, are associated with increased risk of in-hospital mortality. More well-designed studies, that take into account the adjustment for confounders, are required to confirm these associations further. The findings also underscore the need for better evaluation of renal function in diabetic patients with concurrent sepsis.

## Supplementary Information

**Additional file 1: Table S1.** Search strategy for identification of studies to be included in the review. **Table S2.** Author’s judgements about study quality using the adapted Ottawa-Newcastle Risk of Bias Assessment tool. **Table S3.** Author’s judgements about study quality using the adapted Ottawa-Newcastle Risk of Bias Assessment tool.

## Data Availability

The datasets used and/or analyzed during the current study are available from the corresponding author on reasonable request.

## Abbreviations

MeSH: Medical subject heading
RR: Relative risk
WMD: Weighted mean difference
CI: Confidence intervals
PMN: Polymorphonuclear
